# Micro-RNAs, the Cornerstones of the Future of Radiobiology in Head and Neck Cancers?

**DOI:** 10.3390/curroncol29020069

**Published:** 2022-02-02

**Authors:** Camil Ciprian Mireștean, Roxana Irina Iancu, Dragoș Petru Teodor Iancu

**Affiliations:** 1 Department of Oncology and Radiotherapy, University of Medicine and Pharmacy of Craiova, 200349 Craiova, Romania; mc3313@yahoo.com; 2 Department of Surgery, Railways Clinical Hospital, 700506 Iasi, Romania; 3 Department of Oral Pathology, Faculty of Dentistry, “Gr. T. Popa” University of Medicine and Pharmacy, 700115 Iasi, Romania; 4 Clinical Laboratory Department, “St. Spiridon” Emergency Hospital, 700111 Iasi, Romania; 5 Department of Radiotherapy, Regional Institute of Oncology, 700483 Iasi, Romania; dt_iancu@yahoo.com; 6 Department of Medical Oncology and Radiotherapy, Faculty of Medicine, “Gr. T. Popa” University of Medicine and Pharmacy, 700115 Iasi, Romania

**Keywords:** microRNAs, radiobiology, head and neck cancers, chemotherapy, radiosensitivity, radioresistance

## Abstract

Even though it is only the 6th most common malignancy at the modal level, head and neck cancers are distinguished by a considerable treatment failure rate, especially by locoregional recurrences, the intrinsic tumor radioresistance being one of the causes of this phenomenon. The efforts of radiobiological research of these cancers are oriented towards the identification of biomarkers associated with radioresistance and radiosensitivity in order to modulate the treatment so that the therapeutic benefit is maximum. Micro-RNAs (miRNAs, miRs), small single-stranded non-coding RNA molecules are currently being extensively evaluated as potential biomarkers in numerous diseases, including cancer. The evaluation of the potential of miRNAs to modulate or predict radiosensitivity or radioresistance, to anticipate the risk of recurrence and metastasis, and to differentiate different tumor subtypes is based on multiple mechanisms by which mRNAs control proliferation and apoptosis and interact with cell cycle phases or act as oncogenes with the potential to influence invasion promotion or tumor suppression. A refinement of radiosensitivity based on miRNAs with clinical and radiobiological application in head and neck cancers can lead to a personalization of radiotherapy. Thus, a miRNA signature can anticipate the risk of toxicity associated with chemoradiation, the possibility of obtaining locoregional control after treatment, and the recurrence and distant metastasis risk. The potential of miRNAs as an intrinsic predictor of sensitivity to chemotherapy may also guide the therapeutic decision toward choosing an escalation or de-escalation of concurrent or sequential systemic treatment. The choice of the irradiated dose, the fractional dose, the fractionation scheme, and the refining of the dose-volume constraints depending on the radiosensitivity of each tissue type estimated on a case-by-case basis by miRNAs profile are possible concepts for the future radiotherapy and radiobiology of head and neck cancers.

## 1. Introduction

Head and neck cancers are the 6th most common malignancy worldwide and squamous cell carcinoma of the head and neck (HNSCC) is currently the most common malignant tumor of the head and neck and accounts for approximately 90% of head and neck cancers. With an annual incidence of over 500,000 new cases worldwide, the severity of HNSCC is given by the unfavorable prognosis, the 5-year survival being about 40% even if maximum treatment is administered [1,2,3]. The pattern of therapeutic failure is mainly locoregional recurrence which occurs in 15–50% of cases, but also distant metastasis can be a cause of disease progression [4]. Resistance to oncological therapies (chemotherapy, radiotherapy, molecular therapy, and immunotherapy) is the main pathophysiological phenomenon that underlies the therapeutic failure of this type of cancer. Radiotherapy is part of the adjuvant or definitive treatment of HNSCC as a single treatment or in combination with therapies with synergistic and radiosensitizing potential, such as chemotherapy or targeted molecular therapy. Although the combination of radiation therapy with chemotherapy demonstrates a potential benefit on tumor control, the toxic effects associated with concurrent treatment are severe and may alter the quality of life (QoL) or may even limit the survival of these patients [5,6]. In this context, the identification of agents that increase tumor radiosensitivity could lead to increased local control rates without increasing the rate of toxicity. Even if radiosensitizing agents are not used, there are significant variations in the tumor response to irradiation, modulated by intrinsic factors that modulate radiosensitivity. Protein-encoding genes can modulate the response of tumor and normal tissues to irradiation, but direct gene manipulation is difficult [2,7]. MiRNAs, a class of small endogenous non-coding RNA, generally composed of 22 nucleotides, can provide new horizons in the control of intrinsic radiosensitivity by post-transcriptional regulation of gene expression. It has been shown that miRNA overexpression or knock-down may both alter radiosensitivity of a tumor or normal tissue [7,8,9]. Thus, evaluation of these miRNAs’ expression can predict DNA damage or lead to cell-cycle checkpoint manipulation. Knowledge of each miRNA role may result in the design of radiobiological models based on miRNAs with direct clinical applications [8].

## 2. Aim of Study

Without intending to study the depth of the mechanisms of involvement of each miRNA in modulating radiosensitivity, we want to provide a starting point for radiation oncologist clinicians with an interest in translational research in understanding and applying in clinical trials data obtained from fundamental miRNA research in head and neck cancers. By presenting epidemiological and etiopathogenic data, we wish to provide a miRNA-based bridge to understanding the different response to irradiation with a special focus on differentiating HPV+ and HPV− subtypes from oropharyngeal cancers. Last but not least, the current context created by the COVID-19 pandemic with consequences in the rapid implementation of hypofractionation schemes simultaneously with the large-scale implementation of immunotherapy having potentially synergistic with irradiation requires an in-depth understanding and refinement of the head and neck cancers radiobiology, beyond the basics of the linear quadratic model.

## 3. Head and Neck Cancers: Epidemiology, Etiology, and Pathogenesis

With an incidence of 550,000 cases annually, head and neck cancers represent the 6th neoplasm as the worldwide incidence. The higher death rate (approximately 300,000 per year) is a reason why these cancers require special attention in order to establish measures in order to reduce the incidence and mortality by designing prevention programs but also by refining therapeutic protocols. About 90% of head and neck cancers are squamous cell carcinoma (HNSCC) [6,7,8]. Only a third of these patients are diagnosed in the early stages of the disease (T1-2, NO), the 5-year survival rate being about 40–50% for all HNSCC stages. Smoking is considered the major independent risk factor, both for the development of HNSCC and for development of treatment-related complications such as jaw radionecrosis (in the case of patients receiving radiation therapy). Alcohol is also an independent risk factor for both the development of HNSCC and for the deterioration of quality of life if the patient continues to drink alcohol after completing treatment. A detailed analysis of data from clinical trials published between 2006 and 2009 identifies 55% of 654 oropharyngeal cancer patients as human papilloma virus (HPV) positive. HPV infection has become a growing amplitude risk factor especially in the last decades. Without being able to identify a premalignant clinical lesion, the only effective prevention strategies are the sexual education of young people and the vaccination of 12–13-year-old girls. Oral sex is involved in the transmission of HPV subtypes with a high risk of viral carcinogenesis (subtypes 16 and 18), the prevalence of cases of oral cancer and HPV-positive oropharynx increasing especially in developed countries. Premalignant lesions are also a risk factor for HNSCC, dysplasia being identified by biopsy in 25% of cases of leukoplakia and more commonly in erythroplakia, but the association of HPV infection is not synergistic in these cases. Premalignant conditions such as Fanconi’s anemia, ataxia teleangiectasia Bloom’s syndrome, and Li–Fraumeni syndrome are associated with increased rates of HNSCC and their onset at a young age. The involvement of HPV may be also relevant in HNSCC associated with Fanconi anemia [3,10,11,12,13]. Analyzing data from 1990 to 2017, Auperin highlights a decrease in the incidence of nasopharyngeal and laryngeal cancer worldwide and an increase in the incidence of pharyngeal and lip/oral cavity cancers [14]. An increased incidence of HPV etiology in the oral cavity and oropharynx cancers could justify this change in the ratio of head and neck cancer subtypes. The study mentions the consumption of fruits and vegetables but also the vaccination of boys as strategies to reduce the incidence of HNSCC [14].

HNSCC develops from the mucosal epithelium, the pathophysiological process of carcinogenesis passing successively through the stage of hyperplasia, dysplasia, carcinoma in situ, and finally invasive carcinoma. Being a heterogeneous disease, both by different anatomical locations and by etiological factors (viral or carcinogenic), the cells of origin of HNSCC are usually adult stem cells or progenitors. Even if the pluripotent self-renewal stem cell is represented by only 1–3%, cancers caused by this type of cell with the ability to self-replicate when it is transplanted in other tissues is distinguished by resistance to standard treatment. CD44 (a surface receptor for hyaluronic acid and matrix metalloproteinases), ALDH1 (an intracellular enzyme with a role in detoxification), and CD133 (a membrane-spanning protein) are involved in the process of tumor invasion and metastasis, having a decisive role in prognosis [15,16,17].

The increased frequency of synchronous and metachronous cancers, both in different segments of the head and neck and in the lung or esophagus associated with field cancerization have as substrate molecular abnormalities, the carcinogenesis process in the second primary tumor being amplified by smoking. The presence of ALDH1 in the vicinity of vessels justifies the increased risk of metastasis for cases that have high levels of ALDH1 [18,19].

In the case of HPV-negative cancers, the most incriminated carcinogens are polycyclic aromatic hydrocarbons and especially nitrosamines in the case of tobacco exposure, but for situations where the main carcinogen is betel quid or areca nut, the mechanism is not so much studied. A destabilization of the balance between metabolic activation and detoxification of carcinogen-induced DNA damage underlies carcinogenesis in HPV-negative HNSCC. The metabolism of alcohol to acetic aldehyde increases the number of lesions accumulated, so alcohol is a potentiating factor of carcinogenesis [20]. In the case of HPV+ cancers, carcinogenesis begins in the crypts of the palatal and lingual tonsils. Along with the HPV-16 subtype, which is undoubtedly dominant as a risk factor, in a minority of cases HPV-52, HPV-18, HPV-31, and HPV-33 subtypes could also be identified [21].

In the case of the HPV-16 subtype, the E1–E5 genes encode proteins involved in the replication and transcription of the viral genome and the E6 and E7 genes are associated with carcinogenesis. The E6 gene acts through the synthetic protein product on the tumor suppressor p53, degrading it. E7, via retinoblastoma-associated protein (RB1), acts at the nickel control points of the cell cycle. Alteration of RB1 function also has the effect of upregulating the p16 protein, well known as a surrogate biomarker in the identification of HPV− or HPV+ oropharyngeal cancer discrimination. All these totally different mechanisms in the case of HNSCC HPV− and HPV + highlight that we can talk about two different diseases not only as an etiology, but also as a pathophysiological mechanism of carcinogenesis [22,23].

## 4. MicroRNA Involvement in Head and Neck Cancer Development

Human papillomaviruses (HPV) are involved in the etiopathogenesis of at least three type of cancers (cervical, anal, and HNSCC). Although the involvement of HPV infection in the case of cervical cancer is incriminated in almost all cases, there is only a part of head and neck cancers associated with this viral infection. It is obvious that the HPV infection causes the hijacking of the host cell pathways, but the identification of the targets within this cell as well as their contribution to the malignancy process is also of major importance. The Hippo pathway, involved in epithelial homeostasis, is thought to be involved in the carcinogenesis and progression of HPV-induced cancer. Morgan and collaborators propose the activation of the Hippo pathway as a therapeutic target in cancers associated with HPV infection. Serine/threonine-protein kinase 4 (STK4), the master Hippo kinase is identified as low in HPV-associated cancers and HPV-associated proteins E6 and E7 upregulate the miR-18, thus promoting tumor genesis by inhibiting STK4. The suppressive effect of miR-18 on STK4 and indirectly tumor promotion is identified not only in HPV-induced cancers but also in prostate cancer [24,25].

The development of the tumor phenotype is based on alterations in tumor suppressor oncogenes in tumor cells and stromal cells. These phenomena have the effect of transforming the normal epithelium into carcinoma in situ and subsequently into invasive squamous cell carcinoma. Loss of p53 control by viral mutations has the final effect of carcinogenesis by inhibiting apoptosis. In HPV-positive cancers, inhibition of p53 is mediated by the E6 protein characteristic of the HPV16 subtype. Tumor proliferation is also supported by p16 inactivation and overexpression of cyclin D in HNSCC. Epidermal growth factor (EGFR) is involved in the control of multiple pathways that mediate both proliferation and invasion and migration. EGFR also modulates tumor survival and angiogenesis, and EGF-induced STAT3 signaling initiates the transcription of genes that modulate cell growth, survival, and angiogenesis (Cyclyn D1, Bcl-XL, and VEGF, respectively) [25,26,27,28,29,30,31,32]. Progression and metastasis involve the interaction of the tumor with the tumor microenvironment and the remodeling of it and the stromal cells, a cytokine-mediated interaction. Angiogenesis, basement membrane modification, and tumor proliferation are also processes in which cytokines are involved. Infiltrating immune and endothelial cells but also cancer fibroblasts have been associated with the process of distant metastasis. Cancer-associated fibroblasts already shown to be associated with tumor progression have been correlated by Shi et al. with the occurrence of lung metastasis. Evaluated in a preclinical model, TGFβ-enriched fibroblasts and TGFβ activation were correlated with the risk of micrometastases [32,33,34].

The role of miRNA in the carcinogenesis process is both to regulate genes that suppress tumors, their blocking being associated with carcinogenesis, but also to block oncogenes. Deletion of miRNA genes leads to increased oncogenic production, favoring tumor progression [30,32]. Moreover, miR-15a and miR-16-1 are the first miRNAs identified as involved in carcinogenesis, being detected in more than 50% of cases of chronic leukemia. The oncogenic effect of these two miRNAs is mediated by the modulation of the BCL-2 antiapoptotic gene [28,29]. MiR-21 has been shown to be frequently identified in HNSCC and has the role of reducing the expression of PTEN, a modulator of the phosphoinositide 3-kinasePI3K pathway, the most commonly mutated pathway in HNSCC. Another miRNA involved in the carcinogenesis of head and neck cancers is miR-31. It promotes tumor progression and angioneogenesis by activating the hypoxia-inducible factor (HIF) pathway. MiR-375, identified in over 90% of HNSCC, is considered a tumor suppressor [30,31,32]. miR-34a is involved in tumor suppression, its reduced expression being identified in pancreatic, breast, and lung cancer [35,36,37].

MiR-34a demonstrated a significant role in mediating apoptosis, senescence, and related to p53-mediated cell cycle arrest, directly targeting cyclin E2, BCL-2cyclin-dependent kinases (CDK4 and CDK6). MiR-34 family loss in tumors was also associated with tumorigenesis [35,36]. Recent studies demonstrate the potential of miR-34a to modulate tumor growth by denervating the p53-mediated tumor microenvironment [37]. P53 modulates cell survival through indirect control of hypoxia and angiogenesis. Knockdown of endogenous miR-107 may act to amplify inducible factor-1beta hypoxia (HIF-1beta) and overexpression of miR-107 reduces angiosis. MiR-192 and miR-215 are effectors and regulators of p53 and may suppress tumor genesis by cell cycle arrest [38]. No miRNA has been shown to directly modulate capillary tumor extravasation to favor metastasis, but miR-520/373, miR-204, and miR-200 modulate tumor angiosis via TGFβ by tumor-associated fibroblasts (TAFs) [39]. MiR-30a-5p interacts with the MET and EGFR pathways thus being an indirect suppressor of tumor growth [40]. Mammalian target of rapamycin (mTOR) of serine-threonine kinase protein with PI3K/Akt pathway signaling role also has a role in tumor proliferation independent of EGFR and p53. The mTOR pathway can also modulate epithelial-mesenchymal transition (EMT), thus being a promoter of tumor migration. MiR-7, miR-99a, miR-100, and miR-101 are just some of the miRNAs involved in modulating this pathway in cancer [41]. MiRNA-7 inhibits tumor growth and metastasis by targeting the phosphatidylinositol 3-kinase/Akt pathway in hepatocellular carcinoma [42]. Silencing of miR-17-5p can block HNSCC tumor cells in the G2/M phase, thus demonstrating the potential of this miRNA to promote tumor growth and progression [43].

## 5. miRNAs and Cancer-Implications in Clinical Practice—Focus on Radiobiology:

The interest in the value of miRNA in the radiobiology of the future is justified by the vast number of reports that mention the involvement of these small non-coding RNAs both in the development and normal function of organs (brain development and functioning) and in the pathogenesis of diseases such as neuropsychiatric disorders, schizophrenia or bipolar disorder, atherosclerosis, cardiac hypertrophy, and systemic lupus erythematosus. Their huge potential for regulatory molecules opens new horizons in the medicine of the future and in the treatment and diagnosis of many diseases [33,34,44]. The modulation potential of radiosensitivity is given by the involvement of miRNAs in the differentiation and proliferation and cell death. By specifically altering these cellular functions of both cancer and other diseases, miRNA demonstrates its potential to be used as a circulating noninvasive biomarker. Even if the many prognostic and predictive risk factors for the cancer evolution are currently used in daily clinical practice (such as tumor staging according to TNM classification, histological type, degree of cell differentiation, genetic mutations, and expressions of some proteins with biomarker value), a refining of these biomarkers is necessary, especially regarding both the tumor and the normal tissue response to irradiation. Thus, it will be easier to anticipate as accurately as possible the risk of toxicity but also the possibility of tumor control for each individual case [33,44,45]. As early as 25 years ago, the different response to irradiation was considered a consequence of the variation in intrinsic radiosensitivity. Pekkola-Heino and collaborators evaluated the average value of radiosensitivity at 1.9 Gy with variations between 1.0 Gy and 2.8 Gy, finding sensitive differences even between cell populations [46]. Cancers are also among the diseases in which miRNAs are involved both in down-regulation and up-regulation of different genes involved in tumor radiosensitivity by activating mechanisms of radioresistance or radiosensitization. MiRNAs’ impact can be augmented via a carcinogenesis effect exercised by down-regulation of the tumor suppressor gene or by modulating cell proliferation and apoptosis. The large amount of evidence that makes the connection between down-regulation, up-regulation, knockdown, overexpression, or other dysregulation of miRNAs in various cancers justifies the hypothesis that these small single-stranded non-coding RNA molecules will play an essential role in the oncology of the near future. MiRNAs are modulators of radiosensitivity through effects on phenomena such as cell damage repair, apoptosis, and free radical generation [47,48,49].

Mi-R-139-5p is associated with the accumulation of DNA damage caused by irradiation via the methionine adenosyltransferase 2A (MAT2A) gene. Another pathway for modulating DNA damage involved in radiosensitivity is the mutant ataxia-telangiectasia (ATM) and ataxia-telangiectasia (ATR) genes that modulate DNA damage repair via cyclin-dependent kinase (CDK) [41,42]. MiR-16 and miiR-15/ab are involved in the modulation of this pathway. Blocking the formation of CDK/Cyclin complexes limits the transition of the cell from phase G1 to phase S and from phase G2 to phase M. MiR-15 family and miR-449 are involved in G2 and G2/M phase arrest. ATM/P53/P21 is another pathway with cell cycle implications and miR-200c, miR-375, and miR-106b controlled this pathway [50,51,52]. Also worth mentioning is miR-208a, a radiation-induced mi-RNA that can produce radioresistance by activating the AKT/mTOR pathway [50,51,52,53,54]. A direct or inverse correlation between miRNA and specific proteins, determinants of tumor radiosensitivity in breast cancer, has been identified. Among these proteins, we mention proteins that are therapeutic targets in several types of cancers: vascular endothelial growth factor (VEGF) and epidermal growth factor receptor (EGFR), human epidermal growth factor receptor-2 (Her-2/neu) whose status is involved in the molecular classification of breast cancer, and p53, a well-known predictor both of radiosensitivity and of sensitivity to platinum-based chemotherapy. The mechanism of direct and inverse correlation is given by the role of these proteins in regulating the expression of some miRNAs, but miRNAs also have a role in regulating proteins at the post-transcriptional level [55,56]. MiR-18a has the potential to be a radiosensitivity biomarker and an actor that modulates radioresistance, having a suppressive effect on ATM genes, with direct influence on the ability to repair double-strand DNA breaks after irradiation [33,34]. Without considering that we cover the entire vast field of radiosensitivity modulation, the purpose of the example was to highlight the role of miRNAs in the radiosensitivity of one of the most heterogeneous cancers, HNSCC. MiR-218 identified in tumor tissue is associated with unfavorable evolution and cancer progression. In fact, it has been shown that up-regulation of miR-218 is also involved in potentiating irradiation-induced apoptosis and due to this mechanism, miR-218 becomes a potential biomarker of radiosensitivity in cervical cancer. EMT and angiogenesis are regulated in colorectal cancer by miR-218 [57]. MiR-145 expression is associated with radiosensitivity in high-risk human papilloma virus (HPV) associated with cervical cancer, the mechanism being one of synergistic interaction with long non-coding RNA mucosa-associated lymphoid tissue via the lymphoma translocation protein 1 (MALAT1) [58,59,60]. MiR-139 can modulate radiosensitivity by accumulating DNA damage, the mechanism being mediated by the MAT2A gene [29]. The accumulation of radiation-induced DNA damage has as its substrate the inhibition of two mechanisms: non-homologous end joining (NHEJ) and homologous recombination (HR) pathways, methods by which under normal conditions DNA damage is repaired. If the total repair of the lesions has occurred, the cell may die by entering in the apoptosis, suffering a mitotic catastrophe, or it may continue the normal cell cycle [61]. MiR-208a-modulated radiosensitivity has been demonstrated in both preclinical cell and animal models and miR-208a has been associated with radioresistance and proliferation in lung cancer. In patients with non-small cell lung carcinoma (NSCLC), overexpression of hsa-miR-96-5p and hsa-miR-874-3p associated with irradiation may potentiate the tumoricidal effect, the results being similar to the situation where systemic agents targeting HR and NHEJ pathways are added to radiotherapy [50,62]. PTEN down-regulation has as a consequence the activation of the PI3K/AKT pathway, associated with radioresistance and tumor proliferation. MiR-10b reduces the antiproliferative effect of irradiation by activating caspase 3/7 and inhibiting Bcl-2 expression [63,64,65,66]. Thus, activating p-AKT, miR-10b expression reduces the sensitivity of glioblastoma to irradiation promoting proliferation and tumoral invasion [64,65].

## 6. miRNA in Head and Neck Cancers—From the Biomarker of the Future to the Orchestrator of Radiosensitivity

The huge potential of miRNAs to become valuable and accurate biomarkers for diagnostic and prognosis in the future is supported by several basic characteristics: they are rapidly synthesized in a certain clinical situation, have a high specificity, and remain in the environment from which they are identified a long period of time. It is generally considered that there are three types of diseases in which miRNAs have and will have a key role as a biomarker (cardiovascular diseases, infectious diseases, and neoplasms) [57,58,59,60]. Nearly a decade after the launch of the first miRNA panel that opened the horizons for the widespread use of miRNAs in medical practice, there are still problems standardizing and establishing relationships between a miRNA or set of miRNAs and a particular disease. In clinical practice, the first use of miRNA as a biomarker is attributed to Lawrie and collaborators who, in 2008, identified higher levels of tumor-specific miRNAs in patients with large B-cell lymphoma [66,67,68,69,70,71,72].

MiR-138 was associated with the control of transcriptional activity of E-cadherin and elevated levels were negatively correlated with the risk of metastasis. Being commonly reported in HNSCC miR-138, associated with EMT this miRNA is a potential prognostic biomarker for HNSCC [64,65,66,67]. Different results are reported for miR-205-5p; identified in peritumoral tissue, this miRNA is associated with early detection of minimal residual disease involved in tumor development. MiR-205-5p is also considered a tumor suppressor and a limiter of tumor migration and invasion in squamous cell carcinoma of the oral cavity. An association with let-7d of this miRNA may constitute a combined biomarker of survival and prognosis in HNSCC [73,74,75,76,77]. Reduced miR-29c-5p expression is associated not only with the malignant phenotype in HNSCC but also has prognostic and therapeutic implications. Up-regulation of miR-29c-5p is correlated with the arrest of tumor cells in the G2/M phase, being associated with reduced tumor proliferation both in vivo and in vitro. This miRNA proves to be not only a biomarker but also a potential therapeutic target [78].

Intensity Modulated Radiation Therapy (IMRT) in a dose of at least 70Gy in 35 daily fraction, 5 fractions per week over 7 weeks, and concurrent chemotherapy with cisplatin is currently the therapeutic standard for the treatment of locally advanced non-metastatic head and neck cancers. Cisplatin, a platinum-based alkylating agent, has the potential for radiosensitization, being, so far, the backbone of the concurrent association between radiation therapy and systemic therapy. Even if given weekly at a dose of 40mg/m^2^ or 100 mg/m^2^ every 3 weeks, new evidence indicates that a cumulative dose of at least 200 mg/m^2^ is associated with therapeutic benefit. In addition, the stratification of patients with squamous cell carcinomas of the head and neck (HNSCCs) according to human papilloma virus (HPV) status in HPV+ and HPV− tumors showed differences in response to chemoradiation with a higher response rate in non-smokers, HPV+ [79,80,81,82,83,84]. Although the role of induction chemotherapy is still controversial, the triple combination of TPF (docetaxel, carboplatin, and fluorouracil) followed by platinum-based chemoradiation (CCRT) may be considered with benefits in locoregional control for certain categories of patients. Contrary to expectations, trials that proposed de-escalating treatment for oropharyngeal HNSCC HPV + cases were negative. However, some controversial topics exist regarding the concept of de-escalation, requiring a refinement of results. Analyzing the data from a small Italian phase II trial, the significantly lower (50% vs. 83%) OS at 2 years for patients who received cetuximab and not cisplatin suggests that it can be estimated that it is not the reduction of irradiation dose that would be the cause of treatment failure but de-escalation of chemotherapy by substituting cisplatin with cetuximab [85,86,87]. If currently these biomarkers of radiosensitivity have no implications in the clinical decision, the de-escalation of chemo-radiotherapy treatment in certain groups of patients being only the subject of clinical trials, miRNAs open new horizons in customizing treatments based on different radio-chemo-sensitivity [88,89,90].

The COVID-19 pandemic has brought back the need for an irradiation regimen to limit patient and staff exposure during prolonged treatment. Thus, hypofractionated accelerated chemoradiation was evaluated on a batch of 564 patients in the PET NECK study trial. Even if in this pandemic context, a moderately hypofractionated treatment regimen (fractions less than or equal to 2.4 Gy per fraction) is feasible for chemo-irradiation, if hypofractionated regimens are used such as 55Gy in 20 daily fractions, a careful prediction of the benefit and risk in terms of tumor control and the risk of toxicity is necessary. In the context of an accelerated implementation of hypofractionation schemes, the development of new radiosensitivity biomarkers is essential [91,92,93].

Immunotherapy with immune checkpoint inhibitors (ICIs) has recently become the therapeutic standard in recurrent and metastatic HNSCC. The interest for the addition of radiotherapy in order to potentiate the effect of radiotherapy is increased considering the relatively low response rate. The mechanisms involved are multiple, with both theories on the immunostimulatory and immunosuppressive effect of tumor irradiation and tumor microenvironment existing. The implications of other factors including the microbiome in the synergistic effect of radiotherapy and immunotherapy are still little known. In the context of this new possible indication of radiotherapy, the identification of biomarkers both for the response to treatment with ICI or for radiotherapy–immunotherapy combination is one of maximum interest [94,95].

Analyzing data obtained from 515 HNSCC samples and 44 normal tissues, Luo et al. highlights deregulated miRNAs for both positive and negative HPV HNSCC cases [96]. In the patient lot including 97 HPV + patients, 282 miRNAs were identified and after statistical analysis, a 7 miRNA signature was considered proper for its prognostic value. In the lot including 418 HPV− HNSCC patients, among 289 miRNAs, 6 were included in the prognostic signature. Importantly, the signature of miRNAs for the two types of HNSCC is completely different. In the HPV+ group, miRNAs associated with the unfavorable prognosis are negatively correlated with CD8+ T lymphocytes. MiRNAs associated with a better OS have been associated with NK cells and T regulatory cells (Tregs). In the HPV− tumors group, miR-605-5p was associated with CD8 + T cells, activated CD4 T cells (considered tumor suppressors), and M1 macrophages. MiR-135-3p was associated with a better survival and it was negatively correlated with M2 macrophages. The authors also propose a risk score based on miRNA, mentioning the involvement of miRNAs correlated with CD8 + T lymphocytes and NK cells, Tregs, and T follicular helper cells (TFH) in the tumor microenvironment in pre-existing antitumor immune response. Cases of HPV− with a high risk of miRNAs could benefit more from target therapies and HPV+ patients with a low risk of miRNAs could benefit more from immunotherapy. The authors mention metabolic disorder as a possible cause of therapeutic failure in HPV− patients with an increased miRNA risk score [66,67,96].

Resistance to cisplatin is modulated by cancer-associated fibroblasts (CAF) which in turn regulate cell survival and proliferation in head and neck cancers. The mechanism of restoration of miR-196a sensitivity to cisplatin is related to depletion of miR-196a in CAF, and the authors demonstrate the exosomal capacity of miR-196a to be a biomarker of cisplatin sensitivity [97].

Factors involved in HNSCC radioresistance or radiosensitivity are numerous and are generally common to all cancers including traditional variable like cells lesions repair capacity, hypoxia, cell cycle position, and cell growth fraction, but also new “actors” such as hepatocyte growth factor receptor (HGFR) or programmed cell death protein 1 and programmed cell death protein 1 (PD-1) and its ligand, programmed death-ligand 1 (PD-L1) [98,99,100,101]. HNSCC is a malignancy of a special interest for the improvement of therapeutic ratio due to the importance of radiotherapy in both adjuvant treatment and definitive treatment in association with chemotherapy or in particular cases with target therapy. The proximity of radiosensitive organs which are exposed to potentially severe or even life-threatening toxicities by irradiation with tumoricidal doses is a serious argument both for improving the technique and conformity of delivered radiation dose as accurately as possible to target volumes in prescribed isodose but also for improving prediction by radiobiological modeling based on new evidence of radiosensitivity [79,80,81,82,83]. The RAS, EGFR, and PI3K/AKT pathways are involved in this process of modulating tumor radiosensitivity. EGFR is currently a clinically validated therapeutic target by using anti-EGFR monoclonal antibodies (cetuximab) in both metastatic and locally advanced settings but also has prognostic value, increased EGFR expression being associated with radioresistance and therapeutic failure. The TP53 suppressor gene, a regulator of genome stability and consequently a manipulator of DNA degradation, is mutant in 40–70% of HNSCC and thus inactivates the product or protein with effect on the cell cycle. Tumor hypoxia induces neovascularization and, by modulating the response to DNA damage, indirectly influences intrinsic radiosensitivity [91,92]. The mutation in the TP53 gene leads to the inactivation of its protein (p53) product and the change in p53 leads to an impaired ability to stop the cell cycle and inhibit apoptosis. Hypoxia-induced neovascularization and epithelial-mesenchymal transition (EMT), a phenomenon that modulates invasion and resistance to apoptosis, are also involved in tumor radioresistance in HNSCC. Cancer stem cell (SCC) populations, by their ability to renew and proliferate indefinitely and the potential for differentiation, are also associated with radioresistance [102,103,104]. A signature consisting of 4 and 12 miRNAs associated with TP53 were identified in HNSCC as being correlated with non-recurrent survival and cancer-specific survival, respectively. MiR-96-5p is overexpressed in tumors that show a p53 mutant, being involved in chemoresistance and radioresistance by activating the PI3K-AKT pathway [105,106,107].

The role of miRNAs in HNSCC radioresistance phenomena has been demonstrated for a group of miR-16, miR-29b, miR-1254, and miR-150 up-regulated miRNAs and the down-regulated let-7e miRNA for the situation where radiosensitivity is not mediated by ATM gene [79,81,89]. MiR-196a and miR-1323 are considered to have oncogenic potential and down-regulation of miR-205 and up-regulation of miR-96 have been associated with radioresistance but through different signaling pathways. Of note is the different effect that the same miRNA has in two different anatomical localizations of cancer for the same histopathological tumoral type. The low level of miR-203 predicts an unfavorable prognosis and early recurrence after radiotherapy in laryngeal cancer and the same miRNA is associated with radiosensitivity in nasopharyngeal cancer cell populations [66,73,89,90]. Given the need for a validated biomarker to be accessible and evaluable in dynamics, the conceptual use of miRNAs as biomarkers obtained from body fluids is a goal of translational research in personalized medicine and particularly in oncology. One of the most studied miRNAs that can be obtained from serum, plasma, or saliva is miR-21. In head and neck cancers, the high expression of this miRNA is associated with an unfavorable prognosis, the risk of distant metastasis. As demonstrated by a study that included 50 HNSCC patients, elevated postoperative miR-21 levels were associated with unfavorable 1-year survival and this observation offers the opportunity for miRNAs to be used as biomarkers in head and neck cancer surgery [89,107]. Down-regulated serum miR-9 has been associated with recurrence and metastasis in nasopharyngeal cancer and miR-31 is involved in the regulation of the hypoxia pathway, a well-known radioresistance factor. A low level of miR-31 in the blood was also observed in nasopharyngeal cancer compared to the level of this miRNA in normal nasopharyngeal tissues. HPV status with an essential role in radiosensitivity of head and neck cancers can be differentiated based on miRNA (miR-9, miR-122, miR-124, miR-134) in p16 positive and negative, as was demonstrated by Salazar-Ruales et al. and Wan et al. [97,105,106,108,109,110]. In oral cancer, miR-802 was identified as down-regulated in approximately 60% of cases using this level of miRNA in normal tissue as a reference. For a relatively “orphan” tumor subtype in terms of systemic treatments with curative potential (cystic adenoid carcinoma of the head and neck), it has been shown that high levels of miR-374c are associated with reduced recurrence rate and miR-21 inhibitors in association with sinvastatin have shown antiproliferative potential in lung metastases of cystic adenoid carcinoma of the salivary glands [89,97,111,112]. 

Without proposing to include the vast number of research papers that analyze the involvement of miRNAs in the radiosensitivity of head and neck cancers, we present in a table the main studies in the field, also mentioning the mechanism of action, binding site, and the radiosensitizing or the radio-resistance augmentation effect for each mentioned miRNA. (Table 1).

## 7. Conclusions

MiRNAs are currently intensively evaluated as potential biomarkers in numerous diseases and cancers. Determining the ability of miRNAs to modulate or predict radiosensitivity and resistance, to anticipate the risk of recurrence and metastasis, and to differentiate different tumor subtypes is based on multiple mechanisms by which mRNAs control proliferation and apoptosis, interact with cell cycle phases, and mechanisms by which miRNAs act as oncogenes with the potential to influence invasion promotion or tumor suppression. A refining of biomarkers of radiosensitivity miRNAs with clinical application in head and neck cancers can lead to a personalization of radiotherapy by anticipating the risk of toxicity, locoregional tumor control, recurrence, and metastasis. The potential to be an intrinsic predictor of sensitivity to chemotherapy may also guide the decision to choose an escalation or de-escalation of concurrent or sequential systemic treatment. Choosing the total irradiation dose, the dose per fraction, the fractionation scheme, and refining the dose-volume constraints according to the radiosensitivity of each tissue type estimated on a case-by-case basis by miRNAs profile are possible concepts and may be the basis for radiotherapy customization and radiobiology of head and neck cancers in the near future. miRNAs will be able to answer questions about the groups that will benefit from de-escalation of treatment and miRNA signatures could stratify patients who will benefit from hypofractionated radiotherapy and we can anticipate that they will answer the controversies regarding synergistic irradiation with immunotherapy in HNSCC.

## Figures and Tables

**Table 1:** MiRNAs that modulate the radiosensitivity of head and neck cancers [113,114,115,116,117,118,119,120,121,122,123,124,125,126,127,128,129,130,131,132,133,134,135,136,137,138,139,140,141,142,143,144,145,146,147,148,149,150,151,152,153,154,155,156,157,158,159,160,161,162,163].

mi-RNA	Tissue of Origin	Cancer Type	Mechanism of Action	Binding Site	Radiosensitivity	References
miR-296-5p	tissue	larynx	not specified PIN1	PIN1(Lee et al. 2014) [135]	radioresistance	Maia et al. 2015 [113]
miR-324-3p	cells and tissue	nasopharynx	targeting WNT2B	RelA promoter (Dharap et al. 2013) [136]	radiosensitivity	Liu et al. 2017 [114]
miR-203	Cells	HNSCC nasopharyngeal	EMT modulation targeting IL8/AKT	PKCα (Wang et al. 2013) [137]	radiosesitivity	de Jong et al. 2015 [115] Qu et al. 2015 [119]
miR-324-3p, miR-93-3p miR-4501	tissue	nasopharynx	down-regulation/*CDH1,* *PTENP1* and *HSP90AA1*	PEDF (Wang et al. 2017) [138]	radioresistance	Li et al. 2013 [116]
miR-371a-5p, miR-34c-5p and miR-1323	tissue	nasopharynx	up-regulation/*ICAM1,* *WNT2B,* *MYC,* *HLA-F,* and *TGF-β1 pathways*	Xiap (Du et al. 2016) [139] XBP1(Bartoszewska et al. 2019) [140] BMP4 and SMAD4; PRKDC (Xie et al. 2020) [141]	radioresistance	Li et al. 2013 [116]
miR-27a-3p	cells	HPV+ HNSCC	up-regulation/DGCR8/miR-27a-3p/SMG1 axis	FBXW7 (Lu et al. 2021) [143]	radiosensitivity	Long et al. 2021 [117]
miR-106a	cells	HPV+ HNSCC	up-regulation/DGCR8/miR-106a/RUNX3 axis	L10; ASK1 (Sharma et al. 2020) [144] (Hong et al. 2018) [145]	radiosensitivity	Zhang et al. 2020 [118]
miR-375	tissue	oropharyngeal	targeting IGF-1R/cycle arrest in G0/G1 phase, increases apoptosis	YBX1 (Liu et al. 2016) [146]	radiosensitivity	Zhang et al. 2017 [120]
miR-9	cells tissue, cells, saliva	HPV + HNSCC	inducing M1 macrophage polarization via down-regulation of PPARδ by targeting KLF5, positively regulates the expression of Sp1	cyclin D1 and Ets1 (Zheng et al. 2013) [147]	radiosensitivity radioreistance	Tong et al. 2020 [164] Citron et al. 2021 [134]
miR-210	cells	HNSCC	modulation of hypoxia	MNT (Zhang et al. 2009) [148]	radioresistance	Gee et al. 2010 [121]
miR-196a	tissue and cells	HNSCC	suppressing annexin A1 /induce EMT	SNP (Wang et al. 2012) [149]	radioresistance	Suh et al. 2015 [122]
miR-24	tissue and cells	Larynx SCC	targeting X-linked inhibitor of apoptosis protein	dihydrofolate reductase gene (Mihsra et al. 2007) [150]	radiosensitivity	Xu et al. 2015 [165]
miR-495	tissue and cells	nasphparynx	targeting GRP78 to regulate EMT	Sox9 (Lee et al. 2014) [151]	radiosensitivity	Feng et al. 2018 [124]
miR-150	cells	nasopharynx	targeting glycogen synthase kinase-3β	Rab1a and Rab31 (Liu et al. 2015) [152]	radiresistance	Huang et al. 2018 [125]
miR-205	cells	nasopharynx	directly targeting PTEN	ErbB3; VEGF-A (Wu et al. 2009) [153]	radioresistance	Qu et al. 2012 [119]
miR-23a	cells	nasopharynx	targeting IL-8/Stat3 pathway	GJA1; p53 (Gindin et al. 2015) [154]	radiosensitivity	Qu et al. 2015 [126]
miR-24	tissue and cells	nasopharynx	inhibits Jab1/CSN5 translation	dihydrofolate reductase gene (Mihsra et al. 2007) [150]	radiosensitivity	Wang et al.2016 [127]
miR-494-3p	cells	oral cavity SCC	downregulation of Bmi1 pathway	SIRT3 (Zeng et al. 2021) [156]	radiosensitivity	Weng et al. 2016 [166]
miR-375	tissue	oral cavity SCC	targeting insulin-like growth factor 1 receptor	KIT; JAK2 (Gyvyte et al. 2020) [157] (Ding et al. 2010) [158]	radiosensitivity	Zhang et al. 2017 [120]
miR-182-5p	cells	HNSCC	radiation-induced antioxidant effect through SESN2	STARD13 (Wu et al. 2021) [159]	radiosensitivity	Lin et al. 2021 [128]
miR-503	cells	HNSCC	inhibition of WEE1	CUGBP1; CCND1; VEGF; E2 F3 (Cui et al. 2021) (Xu et al. 2013) (Ikari et. al 2017) [160,161,162]	radiosensitivity	Ma et al. 2017 [129]
miR-150	cells	nasopharyngeal	targeting glycogen synthase kinase-3β	MALAT1 (Liu et al. 2021) [163]	radioresistance	Huang et al. 2018 [125]
miR-138-1-3p	cells	nasopharynx	EMT and the JAK2/STAT3 signaling pathway	CRIPTO (Du et al. 2021) [130]	radiosensitivity	Du et al. 2021 [130]
miR-195-3p	tissue and cells	nasopharynx	inhibits cyclin dependent kinase 1	CDK1 (Xie at al. 2021) [131]	radiosensitivity	Xie at al. 2021 [131]
miR-19b-3p	cells	nasopharynx	activating the TNFAIP3/NF-κB axis	TNFAIP3 (Huang et al.) 2016 [132]	radioresistance	Huang et al. 2016 [132]
miR-BART4	tissue and cells	nasopharynx	targeting PTEN	PTEN (Wu et al. 2018) [133]	radioresistance	Wu et al. 2018 [133]
miR-96-5p	cells	HNSCC	PI3K-Akt signaling pathway	PTEN (Vahabi et al. 2019) [105]	radioresistance	Vahabi et al. 2019 [105]
